# Parkinson’s disease therapy: what lies ahead?

**DOI:** 10.1007/s00702-023-02641-6

**Published:** 2023-05-05

**Authors:** Andreas Wolff, Nicolas U. Schumacher, Dominik Pürner, Gerrit Machetanz, Antonia F. Demleitner, Emily Feneberg, Maike Hagemeier, Paul Lingor

**Affiliations:** 1 Department of Neurology, School of Medicine, Klinikum rechts der Isar, Technical University of Munich, Ismaninger Straße 22, 81675 Munich, Germany; 2 German Center for Neurodegenerative Diseases (DZNE), Munich, Germany; 3 Munich Cluster for Systems Neurology (SyNergy), Munich, Germany

**Keywords:** Parkinson’s disease, Disease modification, Symptomatic treatment, Healthcare, Therapy development

## Abstract

The worldwide prevalence of Parkinson’s disease (PD) has been constantly increasing in the last decades. With rising life expectancy, a longer disease duration in PD patients is observed, further increasing the need and socioeconomic importance of adequate PD treatment. Today, PD is exclusively treated symptomatically, mainly by dopaminergic stimulation, while efforts to modify disease progression could not yet be translated to the clinics. New formulations of approved drugs and treatment options of motor fluctuations in advanced stages accompanied by telehealth monitoring have improved PD patients care. In addition, continuous improvement in the understanding of PD disease mechanisms resulted in the identification of new pharmacological targets. Applying novel trial designs, targeting of pre-symptomatic disease stages, and the acknowledgment of PD heterogeneity raise hopes to overcome past failures in the development of drugs for disease modification. In this review, we address these recent developments and venture a glimpse into the future of PD therapy in the years to come.

## Introduction

A change in the therapeutic landscape has been achieved for many chronic or previously incurable diseases over the last decade. Antisense oligonucleotide or gene replacement therapy for patients with spinal muscular atrophy (Weiß et al. 2022) and the recent approval of aducanumab and lecanemab for patients with Alzheimer’s disease (Rabinovici 2021; van Dyck et al. 2023) are only selected examples for recent breakthrough developments. In the case of Parkinson’s disease (PD), many disease-modifying therapies (DMTs) have advanced from preclinical into clinical testing, but none of these approaches has yet been able to demonstrate a disease-modifying effect in clinical trials. However, new formulations of established dopaminergic drugs, device-aided applications, and new drug targets for the treatment of motor fluctuations in advanced stages have further improved the care of patients with PD. With nearly 150 registered clinical trials for disease-modifying and symptomatic therapies in the ClinicalTrials.gov database in 2022 (McFarthing et al. 2022), recent advances in biomarker development (Espay et al. 2017b), and increasing implementation of digital tools in the healthcare of PD patients (Hassan et al. 2020), the field of future PD therapies is as vibrant as ever.

In this narrative review, we cover advances in the treatment of motor symptoms in early and later stages of the disease and non-motor symptoms categorized by symptom groups, then discuss recent approaches of disease modification. Thereafter, we address the implementation of biomarker-based or genetic stratification of the PD population and sensor-based clinical monitoring into future treatment routine for PD patients. We emphasize the role of biomarkers in premotor diagnosis and target engagement during clinical trials investigating disease modification. We cover the existing evidence through author knowledge and selective PubMed searches (last search performed January 10, 2023) using the search terms: “biomarker”, “disease modification”, “early diagnosis”, “treatment monitoring”, “motor symptoms”, “non-motor symptoms”, “neurodegeneration”, “Parkinson’s disease”, “precision medicine”, “stratification”, “symptomatic treatment”, “synucleinopathies”, “telehealth”, “wearables”.

## Symptomatic therapy

### Early PD

In current practice, initial PD-specific drug treatment focuses on motor symptoms, mostly by oral administration. Available options include levodopa and dopa decarboxylase inhibitors, non-ergot dopamine agonists, inhibitors of the monoamine oxidase B or, less frequently, amantadine or anticholinergics. Treatment is then tailored to the severity of the symptoms, age, and pre-existing conditions of the patient (Fox et al. 2018). Response to initial therapy is usually satisfactory; nevertheless, further efforts are being made to optimize treatment in early PD stages. Tavapadon, a novel D1/D5 dopamine receptor partial agonist, is currently investigated in several phase 3 studies (NCT04201093, NCT04223193, NCT04542499, NCT04760769) to evaluate effects on motor functions in early and later stage patients with and without motor fluctuations (Table 1). Two earlier phase 2 studies for this compound had been terminated by the sponsor due to insufficient efficacy in advanced stages of PD, but the results still indicated a significant improvement in motor functions in early PD, while being generally well tolerated by patients (Riesenberg et al. 2020). Opicapone, an inhibitor of catechol-O-methyltransferase, already approved by EMA and FDA, is currently investigated in a phase 3 study to further assess possible benefits for patients with early PD without motor fluctuations (NCT04978597). A low-dose combination of two approved compounds, pramipexole and rasagiline (P2B001), is evaluated in patients with early PD. A phase 2 trial indicated a good clinical efficacy without an increased rate of adverse events (Olanow et al. 2017). The results of a phase 3 study are pending (NCT03329508).

**Table 1:** Selection of symptomatic therapies in current clinical trials

	Drug	ID clinicaltrials.gov	Structure/target	Status	Primary outcome	Result in human
Motor symptoms						
Bradykinetic symptoms	Tavapadon	NCT04201093 NCT04223193 NCT04760769	D1-/D5-receptor partial agonism	Phase 3 (active)	MDS-UPDRS parts II + III	Ongoing
		NCT04760769			Safety, QUIP-RS, ESS, C-SSRS, SMWQ, EQ-5D-5L, MDS-UPDRS parts II + III, “ON” time without troublesome dyskinesia in diary	
		NCT04542499			“ON” time without troublesome dyskinesia in diary	
	Opicapone	NCT04978597	Inhibition of catechol-O-methyltransferase	Phase 3 (active)	MDS-UPDRS parts III + IV	Ongoing
	P2B001 (pramipexole and rasagiline)	NCT01968460 NCT03329508	Combination of dopamine agonists	Phase 2 (completed) Phase 3 (active)	MDS-UPDRS parts I-III	Improvement of MDS-UPDRS part III (Phase 2)
	ND0612	NCT02726386	Levodopa/carbidopa subcutaneous	Phase 2 (completed) Phase 3 (active)	Long-term safety + tolerability	Decrease in OFF-time and increase in ON-time without troublesome dyskinesia; infusion site reactions most common side effect
		NCT02577523			Change in daily “OFF” time	
		NCT04006210			“ON” time without troublesome dyskinesia in diary	
	ABBV-951	NCT04380142	Foslevodopa/foscarbidopa subcutaneous	Phase 3 (completed)	“ON” time without troublesome dyskinesia in diary	Decrease in OFF-time and increase in ON-time without troublesome dyskinesia
	IPX-066	NCT00974974	Extended-release levodopa/carbidopa	Phase 3 (completed)	“OFF” time during waking in diary	Reduction of OFF state and dyskinesia
	IPX-203	NCT03670953 NCT03877510	Extended-release levodopa/carbidopa	Phase 3 (completed)	“Good ON” time in 3-day diary Safety	Reduction of OFF state
	WD-1603	NCT05036473	Extended-release levodopa/carbidopa	Phase 2 (active)	MDS-UPDRS parts II + III	Ongoing
	DopaFuse	NCT04778176	Continuous oral levodopa/carbidopa	Phase 2 (completed)	Plasma levodopa concentration	Decrease in OFF-time and increase in ON-time without troublesome dyskinesia
	LECIG	NCT05043103	Levodopa–carbidopa–entacapone jejunal	Observational (active)	Change in OFF-time, ADL, LEDD, CGI, use of PD medication, treatment satisfaction, safety	Ongoing
	MRgFUS	NCT03454425 NCT03964272	STN unilateral/bilateral	NA (active)	Safety, MDS-UPDRS	Improvement of MDS-UPDRS part III (unilateral)
		NCT02263885 NCT03319485	GPI unilateral	NA (completed)	Safety, UDysRS, MDS-UPDRS	Improvement of dyskinesia scores and MDS-UPDRS part III
		NCT04996992 NCT04728295	PT unilateral/bilateral	NA (active)	MDS-UPDRS (Safety)	Improvement of multiple symptoms
Dyskinesia	Mesdopetam	NCT04435431	Dopamine D3 receptor antagonism	Phase 2 (completed)	“ON” time without troublesome dyskinesia in diary	Ongoing
	NLX-112	NCT05148884	5-HT_1A_ receptor agonism	Phase 2 (active)	Safety and tolerability	Ongoing
	Buspirone/zolmitriptan (JM-010)	NCT03956979 NCT04377945 NCT02439203	5-HT_1A_ and 5-HT_1B/D_ receptor agonism	Phase 2 (active)	UDysRS	Ongoing
	AV-101/L-4-chlorokynurenine	NCT04147949	Antagonism at NMDA receptor	Phase 2 (active)	UDysRS	Ongoing
	CPL500036	NCT05297201	PDE_10_A inhibition	Phase 2 (active)	UDysRS	Ongoing
	Dipraglurant	NCT04857359	Negative allosteric modulator of mGluR5	Phase 2 (active)	UDysRS	Ongoing
Tremor	Suvecaltamide	NCT05642442	T-type calcium-channel modulation	Phase 2 (active)	Essential Tremor Rating Scale	Ongoing
	MRgFUS	NCT01772693	VIM unilateral	NA (completed)	Safety	Improvement of tremor scores (62%) in tremor-dominant PD patients
Prevention of falls	Rivastigmine	NCT04226248	Inhibition of acetylcholinesterase	Phase 3 (active)	Fall rate	Ongoing
	TAK-071	NCT04334317	allosteric modulator of the muscarinic M_1_ receptor	Phase 2 (active)	2-min dual-task walking test Plasma concentration	Ongoing
	Pirepemat	NCT05258071	modulating cortical catecholaminergic levels	Phase 2 (active)	Fall rate	Ongoing
Non-motor symptoms						
Depression and anxiety	Nortriptyline vs. escitalopram	NCT03652870	Tricyclic antidepressant vs. selective serotonin reuptake inhibitors	Phase 3 (active)	BDI-II	Ongoing
	Vortioxetine	NCT04301492	Agonism 5-HT_1A_ receptor, (partial) antagonism 5-HT_3_, 5-HT_7_, 5-HT_1D,_ 5-HT_1B_ receptor and inhibition of 5-HT-transporter	Phase 4 (active)	Safety + tolerability	Ongoing
	Psilocybin	NCT04932434	Agonism 5-HT_2A_ receptor	Phase 2 (active)	Safety + tolerability, treatment satisfaction	Ongoing
	Ketamine	NCT04944017	Different structures, e.g., NMDA/AMPA receptors and opioid system	Phase 2 (active)	MADRS	Ongoing
	Pimavanserin	NCT03482882	Inverse agonism/antagonism 5-HT_2c_ receptor	Phase 2 (completed)	HAMD-17	Significant improvement of depressive symptoms
	Multi-Strain Probiotic	NCT03968133	Probiotic effects, mechanisms unclear	Phase 2 (active)	PAS	Ongoing
		NCT05568498			IDS-C	
Psychosis	Pimavanserin	NCT00477672 NCT00658567 NCT01174004	Inverse agonism/antagonism 5-HT_2c_ receptor	Phase 3 (completed)	SAPS-H + D, SAPS-PD	Improvement and prevention of relapses of PD psychosis
	Pimavanserin vs. quetiapine	NCT04373317 NCT05590637		Phase 4 (active)	CGI-I Psychosis, NPI-Q H + D subscore	Ongoing
Impulse control disorders	Pimavanserin	NCT03947216	Inverse agonism/antagonism 5-HT_2c_ receptor	Phase 2 (active)	QUIP-RS	Ongoing
Hallucinations	Ondansetron	NCT04167813	Antagonism of 5-HT_3_ receptors	Phase 2 (active)	Hallucinations	Ongoing
Cognitive impairment	NYX-458	NCT04148391	Modulation of NMDA receptor	Phase 2 (active)	Safety + tolerability, NPI-12, S-STS, MDS-UPDRS part IV	No significant improvement in cognitive functions
	DAAOI-P	NCT04470037	Modulation of NMDA receptor	Phase 2 (active)	Changes in gait, neuropsychiatric symptoms, MDS-UPDRS	Ongoing
	SAGE-718	NCT05318937	Modulation of NMDA receptor	Phase 2 (active)	WAIS-IV Coding Test Score	Ongoing
	CST-103, CST-107	NCT04739423	Agonism at the beta-2-adrenoreceptor	Phase 2 (active)	FERT, DCFS	Ongoing
	CST-2032, CST-107	NCT05104463	Agonism at the beta-2-adrenoreceptor	Phase 2 (active)	Safety + tolerability	Ongoing
	Istradefylline	NCT05333549	Antagonism at the adenosine A2A receptor	Phase 2 (active)	Card Sort test	Ongoing
	Sulforaphane	NCT05084365	Antioxidative effects	Phase 2 (active)	MCCB composite score MDS-UPDRS	Ongoing
	ANAVEX2-73	NCT03774459 NCT04575259	Modulation of the sigma-1-receptor	Phase 2 (completed)	COGDRAS continuity of attention, safety + tolerability	Improvement in motor and cognitive function
	GRF6021	NCT03713957	Human plasma protein fraction	Phase 2 (completed)	Safety + tolerability	Small improvement in Montreal Cognitive Assessment and PDQ-39 quality of life measure
	Ceftriaxone	NCT03413384	Third-generation cephalosporin	Phase 2 (active)	ADAS-Cog Score	Ongoing
	Doxycycline	NCT05492019	Tetracyclic antibiotic	Phase 2 (active)	MDS-UPDRS	Ongoing
	Intranasal insulin	NCT04251585	Peptide hormone	Phase 2 (active)	Safety + tolerability	Ongoing
Constipation	Pyridostigmine	NCT05603715	Inhibition of acetylcholinesterase	Phase 2 (active)	Spontaneous bowel movements	Ongoing
	ENT-1	NCT03781791	Inhibition of aSyn aggregates	Phase 2 (completed)	Safety + tolerability, spontaneous bowel movements	Ongoing
Constipation and disease progression	Fecal microbiota transfer	NCT05204641	Probiotic effects, mechanisms unclear	Phase 2 (active)	MDS-UPDRS part III	Ongoing
	Bifidobacterium triple viable capsules	NCT04871464	Probiotic effects, mechanisms unclear	Phase 4 (active)	MDS-UPDRS parts II + III	Ongoing
	Lactobacillus acidophilus probiotic	NCT05576818	Probiotic effects, mechanisms unclear	Phase 3 (active)	MDS-UPDRS	Ongoing
Sleep	Suvorexant	NCT02729714	Antagonism at orexin receptor	Phase 4 (active)	Sleep efficiency (polysomnogram)	Ongoing
	Continuous subcutaneous apomorphine	NCT02940912	Agonism at D1/D2 receptor	Phase 4 (completed)	PDSS	Improvement in PDSS
	Safinamide	NCT03968744	MAO-B and glutamate release inhibitor	Phase 4 (active)	PDSS	Improvement of motor and non-motor symptoms
Excessive daytime sleepiness	Valiloxybate	NCT05056194	GABA_B_ agonism	Phase 2 (active)	ESS	Ongoing
Overactive bladder symptoms	Effects of solifenacin vs. behavioral therapy	NCT03149809	M3 cholinergic receptor antagonism	Phase 3 (active)	ICIQ-OAB questionnaire	Ongoing
Pain	Injectable apomorphine	NCT04879134	Agonism at D1/D2 receptor	Phase 2/3 (active)	MDS-UPDRS, Likert Visual Analogue Scale	Ongoing
	Opicapone	NCT04986982	Inhibition of catechol-O-methyltransferase	Phase 4 (active)	King’s Parkinson’s Disease Pain Scale	Ongoing
	Cannabis oil	NCT03639064	Modulation of cannabinoid receptors	Phase 2 (active)	Safety + dosis	Ongoing

*NA* not applicable; *ADAS-Cog* Alzheimer’s Disease Assessment Scale—Cognitive subscale, *ADL* Activities of daily life, *BDI-II* Beck Depression Inventory II, *CGI-I (Psychosis)* Clinical Global Impressions (Psychosis) Improvement scale, *COGDRAS* Cognitive Drug Research Computerized Assessment System, *C-SSRS* Columbia-Suicide Severity Rating Scale, *DCFS* Dementia Cognitive Fluctuations Scale, *EQ-5D-5L* EuroQoL 5 Dimension Level Index and Visual Analog Scores, *ESS* Epworth Sleepiness Scale, *FERT* Facial Expression Recognition Task, *GPI* globus pallidus internus, *HAMD-17* Hamilton Depression Scale—17 items, *IDS-C* Inventory of Depressive Symptomatology—Clinician Rated, *LEDD* Levodopa-equivalent daily dose, *MADRS* Montgomery-Åsberg Depression Rating Scale, *MCCB* MATRICS Consensus Cognitive Battery, *MDS-UPDRS* MDS-Unified Parkinson’s Disease-Rating Scale, *NPI-Q H + D* Neuropsychiatry Inventory Questionnaire Hallucinations + Delusions subscore, *NPI-12* Neuropsychiatric Inventory, *PAS* Parkinson’s Anxiety Scale, *PDSS* Parkinson’s disease sleep scale, *PT* pallidothalamic tract, *QUIP-RS* Questionnaire for Impulsive-Compulsive Disorders in Parkinson’s Disease-Rating Scale, *SAPS-H + D* Scale for the Assessment of Positive Symptoms—Hallucinations and Delusions scales, *SAPS-PD* Scale for the Assessment of Positive Symptoms 9-item sum score for Parkinson’s Disease, *SMWQ* Study Medication Withdrawal Questionnaire, *S-STS* Sheehan Suicidality Tracking Scale, *STN* subthalamic nucleus, *UDysRS* Unified Dyskinesia Rating Scale, *VIM* Nucleus ventralis intermedius thalami, *WAIS-IV* Wechsler Adult Intelligence Scale-IV Coding Test Score

### Advanced PD

Advanced-stage PD is characterized by insufficient motor control including motor fluctuations, such as dyskinesia and OFF periods, despite optimized oral dopaminergic medication (Soileau et al. 2022). In everyday clinical routine, the 1-2-5 criteria can be used for the identification of advanced PD patients, referring to the presence of at least 1 h of troublesome dyskinesia, 2 h of OFF periods, and the intake of at least 5 doses of oral levodopa (Antonini et al. 2018b). Mechanistically, variable dopamine plasma levels due to pulsatile oral medication, erratic gastric emptying, and decreasing neuronal buffer capacity can result in motor fluctuations (Antonini et al. 2018a; Dijk et al. 2020). Therefore, symptomatic treatment in the advanced stage needs regular adaptation and new symptomatic options are needed to further improve control of motor fluctuations and increasingly debilitating non-motor symptoms.

As OFF states can occur unheralded and independent from medication intake, on-demand treatment of OFF states is a growing field including the application of new formulations of well-known drugs. Orally inhaled levodopa (CVT-301) recently received marketing authorization by FDA and by EMA as a “rescue agent” for the treatment of patients during recurring OFF states. In addition, a novel sublingual film formulation of apomorphine was recently approved by the FDA for on-demand treatment in OFF state. Clinical studies reported an overall reduction of OFF state and a higher likelihood of achieving ON state for these two formulations, while being generally well tolerated, with cough, upper respiratory infections, and dyskinesia as main side effects of CVT-301 (LeWitt et al. 2019; Farbman et al. 2020) and oropharyngeal side effects, transient nausea, somnolence and dizziness in sublingual apomorphine (Olanow et al. 2020). Oral levodopa in combination with a decarboxylase inhibitor is mainly prescribed in immediate-release (IR) and controlled-release (CR) formulations. Currently, novel extended-release formulations (ER), designed to dissolve at different rates, are investigated. A recently completed phase 3 study with IPX-203 showed a significant, albeit small, reduction of OFF state in treated patients (Hauser et al. 2022). A similar oral formulation, IPX-066, had already received approval by FDA in 2015 after proving to reduce duration of OFF state and the occurrence of dyskinesia in PD patients with motor fluctuations, while being generally well tolerated (Hauser et al. 2013). However, marketing authorization for the EU was withdrawn at the request of the marketing authorization holder in 2019. WD-1603, another oral ER formulation of levodopa/carbidopa, is currently under investigation in a phase 2 study (NCT05036473). Already marketed in Japan, zonisamide has been discussed as suitable add-on therapy especially for patients in advances stages. Zonisamide is a reversible MAO-B inhibitor and T-type calcium-channel antagonist. In RCTs conducted in the Japanese population, it reduced OFF-time without increasing troublesome dyskinesia and a positive effect on tremor has been postulated (Li et al. 2020). Currently, there are no ongoing clinical trials to evaluate efficacy in other populations.

An antagonist of the dopamine D3 receptor, mesdopetam, is currently being evaluated for its effects on dyskinesia in a phase 2b study (NCT04435431). Previous studies in rodent models and a phase 1 study indicated a possible anti-dyskinetic and antipsychotic effect without deterioration of motor functions due to its physicochemical properties similar to a dopamine receptor agonist (Waters et al. 2020; Sjöberg et al. 2021). Targeting serotonergic terminals has been proposed as promising target for the treatment of dyskinesia (Politis et al. 2014; Di Luca et al. 2022), therefore buspirone/zolmitriptan (JM-010), agonists of 5-HT_1A_ and 5-HT_1B/D_, respectively (NCT04377945, NCT03956979), and NLX-112, a 5-HT_1A_ receptor agonist (NCT05148884), are currently investigated. Other oral approaches include AV-101/L-4-chlorokynurenine with antagonistic effects on the NMDA receptor (NCT04147949), CPL500036, a PDE_10_A inhibitor (NCT05297201), and dipraglurant, a negative allosteric modulator of glutamate receptor type 5 (mGluR5; NCT04857359).

Advanced PD leads to an increased impairment of motor and cognitive functions associated with a greater incidence of falls, which may result in injuries, reduced mobility, quality of life, and life expectancy (Fasano et al. 2017). A recent Cochrane review including 156 randomized controlled trials (RCTs) with 7939 participants showed a small to large effect of different forms of physical exercise and interventions on motor function and quality of life in PD patients, i.e., aqua-based, endurance, gait/balance, functional, and multi-domain training (Ernst et al. 2023). As of January 6th, 2023, 95 active or recruiting trials are listed in the clinicaltrials.gov registry to assess the effect of different forms of physical activity or support by assistance devices. Currently investigated pharmacologic approaches for the prevention of falls in PD patients include transdermal rivastigmine, a cholinergic drug (phase 3: NCT04226248), oral TAK-071, an allosteric modulator of the muscarinic M_1_ receptor (phase 2: NCT04334317), and pirepemat, modulating cortical catecholaminergic levels (phase 2: NCT05258071; Rein-Hedin et al. 2021).

For oral treatment of moderate to severe tremor resistant to usual PD medication, suvecaltamide, a T-type calcium-channel modulator, is currently evaluated in a recently initiated phase 2 study (NCT05642442).

Three well-established device-aided therapies have been used in advanced PD since the 1990s and 2000s: deep brain stimulation (DBS), Levodopa–carbidopa intestinal gel (LCIG), and continuous subcutaneous apomorphine infusion (CSAI). The latter two avoid fluctuating plasma drug levels by continuously and directly delivering dopaminergic medication into the small intestine (LCIG) or subcutaneously (CSAI) (Dijk et al. 2020), while DBS directly influences locomotor circuits of the basal ganglia (Okun 2012). Randomized trials have proven safety and significant efficacy on motor fluctuations of these device-aided therapies (Deuschl et al. 2006; Katzenschlager et al. 2018; Olanow et al. 2014; Schuepbach et al. 2013; Williams et al. 2010). Recently, jejunal application of levodopa/carbidopa and entacapone (LECIG) in a 4:1:4 ratio via a portable pump could achieve similar levodopa plasma levels with an intestinal levodopa dosage reduction of 35% compared to LCIG (Senek et al. 2020, 2017). An ongoing observational study (NCT05043103) will provide further long-term outcomes of LECIG in the next years. A main drawback of approved device-aided therapies lies in their invasiveness. In an explorative setting (NCT04778176), oral levodopa/carbidopa delivered continuously via a tooth-attached pump system (DopaFuse) reduced fluctuations of plasma levodopa levels compared to standard levodopa/carbidopa, improved ON-time without severe dyskinesia and OFF-time, while being well tolerated (SynAgile Corporation 2022). In addition, two new subcutaneous application approaches avoid invasive surgery and are potentially reversible (Rosebraugh et al. 2021a). A novel levodopa/carbidopa formulation (ND0612) with optimized aqueous solubility and stability can be delivered via a portable pump system (LeWitt et al. 2022; Olanow et al. 2021). In a 8:1 levodopa/carbidopa ratio, several phase 1 and 2 trials proved stable, dosage-proportional and thereby steerable levodopa plasma levels (LeWitt et al. 2022; Giladi et al. 2021), resulting in significant decreases in OFF-time and ON-time with troublesome dyskinesia, while increasing ON-time without troublesome dyskinesia (NCT02577523; Olanow et al. 2021). Due to the relatively large infusion volume, infusion site reactions (mostly mild and reversible nodules, erythema, hematoma, infection in up to 95% of all patients) are the most common side effects (Olanow et al. 2021; Giladi et al. 2021), resulting in a relevant dropout rate in the 1-year interim analysis of the phase 2 open-label study (NCT02726386; Poewe et al. 2021). A multicenter, double-dummy-controlled phase 3 trial of ND0612 (NCT04006210) is ongoing. In parallel, subcutaneous foslevodopa/foscarbidopa (phosphorylated levodopa/carbidopa prodrugs, ABBV-951) has been evaluated in a multicenter phase 3 trial (NCT04380142; Soileau et al. 2022) and showed significant improvement of motor fluctuations in advanced PD: the mean reduction in OFF-time (1.79 h) and the mean increase in ON-time without troublesome dyskinesia (1.75 h), each compared to oral levodopa/carbidopa alone, were equivalent to the clinical benefits documented for LCIG (respective values 1.91 h and 1.86 h; Olanow et al. 2014). Foslevodopa/foscarbidopa attained high chemical stability and > tenfold aqueous solubility compared to levodopa/carbidopa, thereby avoiding large infusion volumes and enabling high plasma levels of levodopa/carbidopa after subcutaneous application (Soileau et al. 2022; Rosebraugh et al. 2021a, 2021b). Similar to ND0612, mostly mild infusion site reactions were the predominant side effect. Additional phase 3 trials (e.g., NCT04750226, NCT03781167) will provide more safety and efficacy data of foslevodopa/foscarbidopa.

A promising novel approach for advanced and/or tremor-dominant PD patients is MR-guided focused ultrasound (MRgFUS). MRgFUS combines focused high-intensity ultrasound, administered via multiple stereotactic transducers on the skull, with MR thermography and allows non-invasive thermocoagulative target lesioning with submillimeter precision and without classical risks of open surgery (Moosa et al. 2019; Martínez-Fernández et al. 2021; Xu et al. 2021). Unilateral thalamotomy (Nucleus ventralis intermedius thalami; VIM) is the most common MRgFUS target for tremor-dominant, medication-refractory PD. A randomized, sham-controlled, prospective trial (NCT01772693) showed 62% tremor reduction on the contralateral hemibody (Bond et al. 2017). Since 2017, unilateral VIM-thalamotomy is an FDA-approved technique in the United States. The main drawback is the insufficient control of ipsilateral tremor and other PD cardinal symptoms, mainly bradykinesia and rigidity (Martínez-Fernández et al. 2021). Recently, a pilot study showed a maintenance of stable levodopa dosage and sufficient tremor control by unilateral thalamotomy for at least 6 months in early-stage tremor-dominant PD patients, thereby potentially enlarging the target group for thalamotomy (Golfrè Andreasi et al. 2022). Less evidence exists for other targets in MRgFUS. Unilateral MRgFUS of the subthalamic nucleus (STN; subthalamotomy) lead to significant reduction of PD cardinal symptoms in a randomized, sham-controlled, double-blind trial of markedly asymmetric PD patients (NCT03454425) (Martínez-Fernández et al. 2020) and bilateral subthalamotomy is currently investigated in a feasibility study (NCT03964272). Focused ablation of the globus pallidus internus (GPI; pallidotomy) reduced dyskinesia in a pilot study (NCT02263885, Eisenberg et al. 2020) and improved motor function while reducing dyskinesia in patients with motor fluctuations (NCT03319485; Krishna et al. 2023). MRgFUS pallidothalamic tractotomy reduces pallidal overinhibition without ablation of the thalamus, showed promising results in case series (Gallay et al. 2019), and is currently investigated in two open-label trials (NCT04728295, NCT04996992). Mostly transient side effects of MRgFUS comprise paraesthesia, gait disturbance, hemiparesis and—in case of subthalamotomy and pallidotomy—hemichorea, speech and visual deficits (Moosa et al. 2019; Martínez-Fernández et al. 2021). Randomized controlled trials are needed to provide further evidence for clinical outcomes of MRgFUS in PD, including bilateral application.

### Non-motor symptoms

Non-motor symptoms can occur in all stages of PD, have a negative impact on the quality of life, and are associated with poor long-term outcomes (Weintraub et al. 2022). They include neuropsychiatric conditions, autonomic dysfunctions, disorders of sleep, and pain. Non-motor symptoms, especially neurocognitive symptoms, depression and pain, impact quality of life more heavily than motor symptoms (Tarolli et al. 2020). Pharmacological therapies of motor symptoms sometimes present themselves with beneficial effects on non-motor symptoms, therefore sparing adjunctive therapies and reducing polypharmacy. However, non-motor symptoms show a distinct interdependence (Marinus et al. 2018) and therapeutic effects on individual symptoms often overlap with effects on other symptoms of this spectrum. A good example is safinamide. Safinamide, a selective, reversible MAO-B inhibitor that also reduces glutamate release by blocking voltage-dependent sodium channels and modulating calcium channels, was approved in 2015 as add-on therapy to levodopa in advanced PD patients with motor fluctuations. There is evidence to suggest that safinamide also has beneficial effects on sleep, fatigue, mood, and pain (Stocchi et al. 2022) and it is currently investigated in advanced PD patients for its effect on sleep quality (NCT03968744).

Depression and anxiety affect approximately 45% of PD patients, sometimes preceding motor symptoms (Lemke et al. 2004). Treatment includes non-pharmacologic measures, i.e., cognitive behavioral therapy, physical exercise, and first-line pharmacological use of mainly selective serotonin reuptake inhibitors (SSRI), serotonin norepinephrine reuptake inhibitors (SNRI), and tricyclic antidepressants (TCA) with mixed evidence of efficacy in previous RCTs (Bomasang-Layno et al. 2015; Weintraub et al. 2022). TCAs display a disfavored safety profile with special concern for PD patients, because orthostatic hypotension, constipation, confusion, and delirium, particularly in demented patients, are known side effects (Starkstein and Brockman 2017). Non-pharmacological interventions, such as cognitive behavioral therapy and multimodal interventions, e.g., cognitive-based mindfulness therapy, showed promising and robust effects (Starkstein and Brockman 2017). Substances currently evaluated in clinical trials include a comparison of nortriptyline and escitalopram, a TCA and a SSRI (phase 3: NCT03652870), vortioxetine, a modulator of serotonin receptors and transport (phase 4: NCT04301492), psilocybin, a psychoactive alkaloid (phase 2: NCT04932434), and intravenous ketamine (phase 2: NCT04944017).

Psychosis affects up to 60% of PD patients at some point in the course of the disease (Weintraub et al. 2022). Clozapine and quetiapine (off-label) are currently the main drug treatments of PD psychosis due to their low risk of worsening extrapyramidal symptoms (Seppi et al. 2019). Still, medication with atypical antipsychotics may lead to an increased mortality and severe adverse effects in PD patients (Ballard et al. 2015). The oral 5-HT_2c_ receptor inverse agonist/antagonist, pimavanserin, received marketing authorization by the FDA in 2016. Pimavanserin intake resulted in a reduction of psychosis symptoms and prevention of relapse in PD psychosis without worsening of motor or cognitive functions. Notable adverse effects in studies mainly comprised headaches, constipation, edema, hallucinations, and urinary tract infections. Due to QTc prolongation in some patients, additional pharmacovigilance is required (Tariot et al. 2021; Abler et al. 2022; Cummings et al. 2014; Isaacson et al. 2021). Currently, two phase 4 studies for comparison of treatments with quetiapine or pimavanserin are recruiting in the US (NCT04373317, NCT05590637). In addition, there is evidence of improvement of depressive symptoms in a completed phase 2 study (DeKarske et al. 2020). Its effect on impulse control disorders in PD will be assessed in another phase 2 study (NCT03947216). A further phase 2 study assesses the effects of ondansetron treatment, an antagonist at 5-HT_3_ receptors, on hallucinations (NCT04167813).

Cognitive impairment is common in PD with mild cognitive impairment being present in more than 10% of PD patients and increases over the course of the disease. PD with dementia is estimated to develop in more than 70 percent of patients after 10 years of disease progression (Litvan et al. 2011). Current standard for pharmacologic symptomatic therapy primarily consists of cholinesterase inhibitors. Other pathways currently under assessment in clinical trials include modulation of the NMDA receptor (phase 2: NCT04470037, NCT05318937), agonism at the beta-2-adrenoreceptor (phase 2: NCT04739423, NCT05104463), antagonism at the adenosine A2A receptor (phase 2: NCT05333549) and antioxidative effects (NCT05084365). An oral formulation of ANAVEX2-73 (blarcamesine), modulating the sigma-1-receptor, was found to improve motor and cognitive functions in a phase 2 trial and its extension study (NCT03774459, NCT04575259, Barwicki 2022, 2023). Furthermore, infusions of GRF6021, a human plasma protein fraction, displayed satisfactory safety profile and improved quality of live and cognitive functions (phase 2: NCT03713957, Rawner 2021).

Constipation is an early and common non-motor symptom in PD. ENT-1, an oral compound inhibiting formation of α-synuclein aggregates, reduced constipation and additionally improved cognitive and psychosis-related outcome measures (Camilleri et al. 2022). Further currently assessed substances for constipation include pyridostigmine (NCT05603715) and probiotic interventions (NCT05204641), also evaluating the potential impact of the latter on disease progression or depression and anxiety (NCT03575195, NCT03968133, NCT04871464, NCT05568498, NCT05576818).

After improving insomnia in patients with Alzheimer’s disease (Herring et al. 2020), orally administered suvorexant, inducing antagonism at the orexin receptor, is investigated for its effect on insomnia (NCT02729714). In addition, continuous subcutaneous application of apomorphine was shown to improve sleep disturbances in PD patients with motor fluctuations (Cock et al. 2022). Oral GABA_B_ agonist valiloxybate is assessed for possible improvements in excessive daytime sleepiness and sleep quality with already promising results in healthy subjects (NCT05056194, Xiang and Rappaport 2021).

Due to the complex involvement of the dopaminergic system in pain, subcutaneous apomorphine (NCT04879134) or oral opicapone (NCT04986982, Chaudhuri et al. 2022) will be investigated for the treament of pain in PD patients. As previous studies in PD could not establish a significant effect, another phase 2 study is evaluating the use of cannabis oil preparations in PD patients (NCT03639064).

## Disease modification

Although symptomatic treatment has strikingly improved over the last decades, DMT bear the hope to modulate disease progression at its roots and would therefore alter our therapeutic landscape significantly. In the following, we will give a brief overview on disease-modifying targets currently investigated and then focus on promising therapies in advanced stages of development (Table 2). Some approaches for disease modification target specific proteins (LRRK2 or GCase) or organelles (mitochondria or lysosomes), while others focus on the reduction of aSyn pathology, broadly accepted to be relevant in the majority of sporadic PD patients, by influencing aSyn-production, turnover, aggregation, cell-to-cell propagation, or else influence downstream mechanisms such as neuronal survival or immunomodulation (Vijiaratnam et al. 2021).

**Table 2:** Selection of disease modification therapies in pipeline

	Drug	ID clinicaltrials.gov	Structure/target	Status	Primary outcome	Result in human
α-synuclein	anle138b	NCT04208152 NCT04685265	Modulates aSyn aggregation	Phase 1 (completed)	Safety/tolerability	Ongoing
	NPT200-11 (UCB0599)	NCT04875962 NCT04658186 NCT05543252	Inhibits dimer formation	Phase 2 (active)	MDS-UPDRS parts I-III, DAT-SPECT (extension study)	Acceptable safety/tolerability profile and PK
	ION464	NCT04165486	Antisense oligonucleotide targeting SNCA mRNA	Phase 1 (active; MSA)	Safety/Tolerability	Ongoing
	Affitope PD01A, PD03A	NCT01568099 NCT02267434	Vaccination against c-terminal epitope	Phase 1 (completed)	Safety/tolerability	Development of oligomer-binding IgGs; reduction of CSF aSyn oligomers
	UB-312	NCT04075318 NCT05634876	Vaccination against c-terminal epitope	Phase 1/2 (active)	Serum/CSF anti-aSyn AB titers	Antibody induction in serum and CSF of healthy participants
	Cinpanemab (BIIB054)	NCT03318523	N-terminal, aggregate-specific antibody	Phase 2 (terminated)	MDS-UPDRS parts I-III	No effect on motor symptoms
	Prasinezumab (PRX002)	NCT03100149 NCT04777331	C-terminal, aggregate-specific antibody	Phase 2 (active)	Time to progression (MDS-UPDRS III decline > 5 points)	Trend to slow decline in motor function (MDS-UPDRS part III)
	MEDI1341 (TAK-341)	NCT04449484 NCT05526391	C-terminal antibody	Phase 1 (completed) Phase 2 (active; MSA)	UMSARS part I	Ongoing
	Lu AF82422	NCT03611569 NCT05104476	C-terminal antibody	Phase 1 (completed) Phase 2 (active; MSA)	UMSARS part I + II	Ongoing
Neuroinflammation	Fasudil	EudraCT: 2021-003879-34 NCT04734379	Rho kinase inhibitor	Phase 2 (active) Phase 2 (active; Tauopathies)	Safety/tolerability	In clinical use with acceptable safety/tolerability profile
	Azathioprine	EudraCT: 2018-003089-14	Peripheral immune system suppressant	Phase 2	MDS-UPDRS gait/axial score	In clinical use with acceptable safety/tolerability profile
	Exenatide	NCT04232969	GLP-1 receptor agonists	Phase 3 (active)	MDS-UPDRS part III	In clinical use with acceptable safety/tolerability profile
Mitochondria	Ursodeoxycholic acid	NCT03840005	Secondary bile acid	Phase 2 (completed)	Safety/Tolerability	In clinical use with acceptable safety/tolerability profile
LRRK2	DNL151 (BIIB122)	NCT05418673 NCT05348785	Oral LRRK2 inhibitor	Phase 2/3 (active)	Time to worsening in MDS-UPDRS parts II + III	Acceptable safety/tolerability profile; robust target and pathway engagement
	BIIB094	NCT03976349	Antisense oligonucleotide	Phase 1 (active)	Safety/tolerability	Ongoing
GBA	PR001 (LY3884961)	NCT04127578	Glucocerebrosidase gene therapy	Phase 1 (active; GBA-PD)	Safety/tolerability (incl. MRI) + AAV9 and GCase immunogenicity in serum + CSF	Ongoing
	Ambroxol	NCT05778617 NCT02941822 NCT05287503	Chaperone of glucocerebrosidase	Phase 3 (active)	MoCA and progression to MCI or dementia	CNS availability; increased GBA and CSF α-synuclein levels
Regenerative or restorative therapies	STEM-PD	NCT05635409	Stem cell-derived dopamine neurons transplant	Phase 1 (active)	Safety/Tolerability	Ongoing
	MSK-DA01	NCT04802733	Stem cell-derived dopamine neurons transplant	Phase 1 (active)	Safety/Tolerability	Ongoing
	AAV2-GDNF	NCT04167540 NCT01621581	Glial cell line-derived neurotrophic factor gene transfer	Phase 1 (active)	Safety/Tolerability	No parenchymal toxicity in follow-up MRIs

*CSF* cerebrospinal fluid; *MSA* Multisystem atrophy, *LRRK2* Leucine-rich repeat kinase 2, *GBA* Glucocerebrosidase, MDS-UPDRS, UMSARS Modified Unified Multiple System Atrophy Rating Scale, *MoCA* Montreal Cognitive Assessment, *MCI* mild cognitive impairment

### Alpha-synuclein

Aggregated aSyn exerts its toxic effects via reducing synaptic vesicles motility, promoting lysosomal and mitochondrial dysfunction, and impairing protein transport from the endoplasmic reticulum to the Golgi apparatus and autophagy (Wong and Krainc 2017). Therefore, DMTs that focus on reducing or mitigating the aSyn burden have been of highest interest. One of the oldest approaches for disease modification by targeting aSyn pathology focuses on specifically engaging aggregated aSyn and interfering with cell-to-cell transmission by immunotherapy. In 2005, first beneficial histopathological effects of immunization with human aSyn have been proposed (Masliah et al. 2005) and many follow-up studies provided evidence that either immunization against aSyn or treatment with aSyn-specific monoclonal antibodies are suitable to reduce phenotypic and neuropathological alterations in in vitro and in vivo PD models (Masliah et al. 2011; Shahaduzzaman et al. 2015; Schofield et al. 2019; Höllerhage et al. 2022). In the case of active immunization (vaccination) two approaches have advanced to clinical trials. On the one hand, PD01A and PD03A both completed phase 1 in PD patients (NCT01568099; NCT02267434) and ACI-7104, an optimized formulation of PD01A, was announced to proceed to phase 2 (AC Immune SA 2021). On the other hand, UB-312, having completed phase 1 (NCT04075318) just recently (Yu et al. 2022), proceeded to phase 2 in patients with α-synucleinopathies (NCT05634876) and will start patient recruitment in 2023. PD01A and UB-312 presumably act in a comparable way by providing a C-terminal epitope and generating an immune response specific against aSyn oligomers. In terms of passive immunotherapy, results have been inconclusive so far. Cinpanemab (BIIB054), an N-terminal, aggregate-specific antibody, was not able to provide evidence for clinical efficacy in phase 2 (change of MDS-UPDRS I-III after 52 weeks; NCT03318523) and its development has been terminated (Lang et al. 2022). Prasinezumab (PRX002), a C-terminal antibody, missed its primary endpoint in the phase 2 trial (change of MDS-UPDRS I-III after 52 weeks; NCT03100149), yet showed a trend to slow decline in motor function (MDS-UPDRS part III). Therefore, efficacy of prasinezumab is currently investigated in the open-label extension of this trial and in an additional phase 2b study over 18 months (NCT04777331) (Pagano et al. 2021, 2022). Two additional antibodies, MEDI1341 (also known as TAK-341) and Lu AF82422 completed phase 1 trials in healthy volunteers and patients with PD (NCT04449484 and NCT03611569, respectively), both being C-terminal and binding monomeric and aggregated aSyn. Both antibodies are currently investigated in phase 2 trials in patients with MSA (NCT05526391, NCT05104476, respectively).

aSyn-selective antisense oligonucleotides have been shown to reduce aSyn expression in a mouse model of PD and enhanced neurotransmitter release (Alarcón-Arís et al. 2018). A phase 1 trial currently investigates intrathecal administration of antisense oligonucleotide ION464 targeting aSyn-coding mRNA (*SNCA* mRNA) in patients with Multiple System Atrophy (MSA), another α-synucleinopathy, and might therefore be potentially relevant for PD patients in the future (NCT04165486).

Very recently, two small molecules with potential to inhibit the formation of presumably toxic aSyn oligomers have been investigated: anle138b, an orally administered diphenyl-pyrazole, potent to modulate aSyn aggregate formation and even to disintegrate aSyn oligomers, has shown a favorable safety and tolerability profile in healthy subjects (NCT04208152; Levin et al. 2022) and has completed the recruitment process in a phase 1 trial in PD patients (NCT04685265), with results expected in 2023. In parallel, NPT200-11, also known as UCB0599, binds to the C-terminus of the aSyn monomer and inhibits dimer formation leading to reduced cortical aSyn pathology and neuroinflammation while improving motor function in a mouse model overexpressing human aSyn (Wrasidlo et al. 2016; Price et al. 2018). Oral UCB0599 has provided reasonable safety/tolerability profile with favorable pharmacokinetics in PD patients (NCT04875962; Smit et al. 2022) and has advanced to a phase 2 trial (NCT04658186) with an extension study planned (NCT05543252).

### Neuroinflammation

A pro-inflammatory immune phenotype, characterized by innate and adaptive immune cell activation, increase of circulating pro-inflammatory cytokines, blood–brain barrier permeability and peripheral immune cell infiltration of the central nervous system has been identified as hallmark of PD. Immunomodulatory or anti-inflammatory approaches may therefore represent promising disease-modifying targets (Tansey et al. 2022; Caldi Gomes et al. 2022). Some anti-inflammatory efforts have failed to provide satisfactory efficacy to date (e.g., NSAIDs (Poly et al. 2019), simvastatin (Stevens et al. 2022), verdiperstat (Biohaven Pharmaceutical Holding Company Ltd. 2021)). Modulation of microglia activation may represent another promising target. Fasudil, a neuroprotective Rho-kinase inhibitor (Tatenhorst et al. 2016) reducing pro-inflammatory cytokines (Zhao et al. 2015) and regulating microglia activation (Zhang et al. 2013), just recently completed recruitment as DMT for amyotrophic lateral sclerosis (ALS, NCT03792490) and will be evaluated in its oral formulation in patients with tauopathies (NCT04734379) and PD patients (EudraCT: 2021-003879-34; authors note). Furthermore, the disease-modifying potential of azathioprine, which reduces T and B lymphocyte proliferation and therefore attenuates inflammatory response, will be evaluated in more rapidly progressing PD patients (EudraCT: 2018-003089-14; Greenland et al. 2020). Patients will be selected by a previously established prognostic model, based on age and clinical evaluation (higher UPDRS-ME axial score and lower animal fluency score; Velseboer et al. 2016). GLP-1 receptor agonists, approved as treatment for type 2 diabetes, have shown promising preclinical results by reducing neuroinflammation (Chen et al. 2018) and decreasing aSyn burden (Zhang et al. 2019). Different GLP-1 receptor agonists are currently evaluated with exenatide being the most advanced. Exenatide-PD3 (NCT04232969) will investigate an extended-release formulation of subcutaneous exenatide over 2 years in PD patients with differences in MDS-UPDRS part III as primary outcome.

### Mitochondria

Two relevant genes for the development of early onset PD are *Parkin* and *PINK1,* and mutations result in recessively inherited PD, both leading to compromised neuronal ability to remove damaged mitochondria (mitophagy), leading to increased amount of dysfunctional mitochondria, release of mitochondrial damage-associated molecular patterns (mitoDAMPs), and neuroinflammation (Borsche et al. 2021). While two DMTs targeting mitochondrial dysfunction (inosine and pioglitazone) did not alter disease progression in PD in phase 2 and 3 trials (NINDS Exploratory Trials in Parkinson Disease (NET-PD) FS-ZONE Investigators 2015; Schwarzschild et al. 2021), ursodeoxycholic acid is currently investigated in a phase 2 trial (NCT03840005). Notably, bile acid tauroursodeoxycholic acid (TUDCA) in combination with sodium phenylbutyrate has been evaluated as neuroprotective agent in Alzheimer’s disease (Becky Gohsler 2021) and was just recently approved by the FDA for patients with Amyotrophic lateral sclerosis (Paganoni et al. 2022).

### Leucine-rich repeat kinase 2 (LRRK2)

Another promising target for DMTs is leucine-rich repeat kinase 2 (LRRK2). The *LRRK2* Gly2019Ser mutation is the most common cause of autosomal-dominant PD and common variants in LRRK2 modulate the risk to develop PD. Inhibitors of LRRK2 promote autophagy (Manzoni et al. 2013), reduce neuroinflammation, and are therefore promising candidates for preventing neurodegeneration (Daher et al. 2015). DNL151 (also known as BIIB122), an orally administered LRRK2 inhibitor, has passed phase 1 and its disease-modifying efficacy is currently evaluated in PD patients with known LRRK2 mutation in up to 180 weeks (NCT05418673) and sporadic PD patients without LRRK2 mutation in up to 144 weeks (NCT05348785). In parallel, BIIB094, an intrathecally administered LRRK2 antisense oligonucleotide, is tested for safety and tolerability in sporadic PD patients in 18 global study centers (NCT03976349).

### Glucocerebrosidase (GBA)

In the European population, the most relevant genetic risk factor for PD are variants of *GBA*, the gene encoding for glucocerebrosidase. Mutations increase PD risk ranging from threefold to 15-fold, depending on the variant (Day and Mullin 2021). While homozygous or compound heterozygous *GBA* mutations lead to the lysosomal storage disorder Gaucher’s disease, ~ 10% of European PD patients harbor *GBA* variants (Skrahina et al. 2021). Mutated glucocerebrosidase disrupts glycosphingolipid homoeostasis, leads to lysosomal dysfunction, and aSyn aggregation (Do et al. 2019). A glucocerebrosidase gene therapy (PR001) administered into the cisterna magna has been of highest interest for treatment of Gaucher’s disease (NCT04411654, NCT05487599), but will also be evaluated in PD patients with at least one known *GBA* mutation (NCT04127578). Efforts to increase the activity of glucocerebrosidase have yielded promising results. Ambroxol, acting as a chaperone of the lysosomal enzyme glucocerebrosidase, displayed good target engagement, increased glucocerebrosidase and aSyn levels in CSF, and lead to improvement in MDS-UPDRS part III in an open-label study in patients with and without *GBA* mutation (NCT02941822). Ambroxol has furthermore advanced into a multicenter, placebo-controlled phase 3 trial in PD patients (NCT05778617).

### Regenerative or restorative therapies

At the borderline between symptomatic and disease-modifying therapies, there are two approaches to counteract or even reverse neuron loss in the brains of PD patients, but without interfering with the underlying pathology. Early works on cell replacement therapies by transplantation of dopaminergic neurons derived from fetal mesencephalon into the putamen displayed promising results including long-term survival of grafted neurons (Olanow et al. 2009) yet double-blind trials showed only mild clinical effects in younger or less severely affected patients (Olanow et al. 2003; Freed et al. 2001). This approach, which relies on fetal tissue, presents many challenges for translation to large patient numbers. Recent approaches try to overcome this by utilizing dopaminergic progenitor cells derived from embryonic stem cell lines as grafts (NCT05635409, NCT04802733). Another approach to prevent loss of dopaminergic terminals in the striatum and neuron loss in the substantia nigra is by administration of neurotrophic factors (e.g., glial cell-derived neurotrophic factor (GDNF), neurturin), known to activate signaling cascades critical for neuron survival and neurite outgrowth (Olanow et al. 2015). However, bioavailability in the brain is a major concern for tropic factors requiring an intrathecal or surgical administration. Previous controlled clinical trials failed to translate promising preclinical data into clinical settings (Lang et al. 2006; Olanow et al. 2015). Currently a GDNF gene transfer approach using adeno-associated viral vectors (AAV2-GDNF) is evaluated in open-label phase 1 trials in PD patients (NCT04167540, NCT01621581) with results indicating an acceptable safety profile of the MRI-guided putaminal infusion (Rocco et al. 2022).

### Trial design and biomarkers

Aside from the selection of interventional targets in representative preclinical models, adequate trial designs with sufficient duration, representative measures of clinical disease progression, and a minimum of confounding by symptomatic effects will help to overcome past failures in disease-modifying trials (Vijiaratnam et al. 2021). One promising trial design is the delayed-start design: in a RCT investigating disease-modifying effect of rasagiline (NCT00256204), patients with PD were randomly assigned to either rasagiline (1 mg or 2 mg) for 72 weeks or placebo treatment for 36 weeks, followed by rasagiline (1 mg or 2 mg) for 36 weeks. This trial setup enables to distinguish between symptomatic effects (difference in symptom severity is only present before switch of the delayed-start group to verum) and disease-modifying effects (differences in symptom severity continue to be present to the end of the trial). Biomarkers for target engagement have been established but are not applied in a wider scale. For example, antibodies against aSyn were shown to reduce unbound serum aSyn by up to 97% (Jankovic et al. 2018) and LRRK2 inhibitor BIIB122 reduced concentrations of LRRK2 and phosphorylation (pT73) of Rab10 (Jennings et al. 2023), a direct substrate of LRRK2. So far, there are no biomarkers of histopathological disease progression available as readout for clinical trials of disease modification; however, promising approaches will be covered in the biomarker section of this article.

## One pill fits all versus precision medicine

In current medical practice, we acknowledge the variability of clinical presentations of PD patients and tailor symptomatic treatment accordingly. Numerous studies attempting to identify PD subtypes have focused on clinical markers, but our increasing knowledge of disease heterogeneity suggests that molecular heterogeneity should be considered as well (Mestre et al. 2021; Espay et al. 2017a). Identification of both clinical and molecular subgroups could enable tailored individual therapeutic solutions (Cholerton et al. 2016; Espay et al. 2017b; Titova and Chaudhuri 2017). Molecular stratification can be performed based on (a) pathogenic mutations that affect a particular molecular pathway, (b) the identification of clusters of common genetic variants in genes associated with specific disease-associated pathways, or (c) biomarkers that indicate the level of function/dysfunction of disease-associated pathways in sporadic PD patients.

Several monogenic causes for PD have been identified (Blauwendraat et al. 2020). These findings have for example implicated dysfunction of mitophagy and autophagy in the pathogenesis of PD (Nguyen et al. 2019; Singleton and Hardy 2019). Mendelian PD is thought to potentially serve as a model for the identification of biomarkers representing underlying pathophysiology and for the development of targeted therapies (Hockey et al. 2015; Mortiboys et al. 2013; Peterschmitt et al. 2022). Efforts like the Rostock International PD (ROPAD) and the LRRK2/Luebeck International PD (LIPAD) studies aim at genetic classification and deep phenotyping of PD patients and healthy carriers of pathogenic variants (Skrahina et al. 2021; Usnich et al. 2021). Initial ROPAD data showed a remarkable genetic diagnostic yield with the identification of disease-associated variants in approximately 14% of 1360 screened PD patients. Variants in *GBA* (in 8.5% of all patients screened), *LRRK2* (3.1%), and compound heterozygous *PRKN* variants (0.8%) were identified most frequently. These patients could be included in clinical trials focusing on genetic subgroups, for example with the LRRK2-Inhibitor BIIB122 (NCT05418673) (see section on “disease modification”).

In most PD patients, however, a disease-causing mutation cannot be found. Nevertheless, common genetic variants modify the risk to develop PD with smaller effect sizes. Identified common variants have linked PD to numerous pathways including lysosomal function, the immune system and metabolism (Fernández-Santiago and Sharma 2022). Polygenic risk scores (PRS) compile common low-risk variants and have been shown to be associated with disease risk, age of onset and disease progression (Dehestani et al. 2021; Paul et al. 2018; Pihlstrøm et al. 2022). For patient stratification, it may also be possible to use pathway-specific PRS, which consist of variants related to particular pathways, such as mitochondrial PRS and autophagy-lysosomal PRS (Bandres-Ciga et al. 2020; Billingsley et al. 2019; Dehestani et al. 2022). While pathway-specific PRS may be indicative of an underlying disease process, it seems more intuitive to use direct markers of current pathway function (e.g., ^31^Phosphorus-Magnetic resonance spectroscopy imaging for mitochondrial dysfunction in PD patients, NCT03815916) to define clinically relevant and mechanistically anchored disease subgroups (Rosen and Zeger 2019; Prasuhn et al. 2020).

Mitochondria-associated blood biomarkers do not necessarily reflect mitochondrial dysfunction in neurons or, more specifically, dopaminergic neurons. Expression patterns often are tissue-specific and mtDNA copy number in blood has been shown to be very variable (Pyle et al. 2016; Davis et al. 2020; Müller-Nedebock et al. 2022). Imaging studies using phosphorus magnetic resonance spectroscopy (31P-MRS) to explore in vivo mitochondrial function in PD patients have shown contrasting results to date (Dossi et al. 2019). A seemingly promising approach is the investigation of skin fibroblasts, which can be directly patient-derived and studied as individual readout in vitro. Using a combination of cellular assays, RNA-sequencing based pathway analysis and genotyping, distinct subgroups of PD patients with mitochondrial and lysosomal dysfunction could be identified (Carling et al. 2020). Other biomarkers related to autophagy and lysosomal function have shown inconsistent results (Xicoy et al. 2019). In sporadic PD, reduced heatshock cognate-70 (Hsc70) levels in peripheral blood mononuclear cells (PBMC) were suggested as a marker of chaperone-mediated autophagy dysfunction, but large-scale studies are still missing (Papagiannakis et al. 2015; Sala et al. 2014). Specific *GBA* variants are associated with variable but consistent reduction in glucocerebrosidase (GCase) activity (Alcalay et al. 2015; Lerche et al. 2021). In sporadic PD, several studies demonstrated a significant reduction of GCase activity while some did not find a difference compared to controls (Atashrazm et al. 2018; van Dijk et al. 2013; Xicoy et al. 2019). Therapies aiming to enhance GCase function in GBA-PD could also be beneficial for sporadic PD patients with reduced GCase activity (Heijer et al. 2021). Inflammatory biomarkers such as IL-6, IL-10, IL-1β, tumor necrosis factor and others have been shown to be increased in blood and CSF of patients with PD (Harms et al. 2021; Qin et al. 2016; Zimmermann and Brockmann 2022). The extent of inflammation is associated with the clinical presentation in patients with LRRK2-associated PD and disease progression in sporadic PD (Brockmann et al. 2017; Williams-Gray et al. 2016). Inflammatory markers in CSF and blood often are not correlated, change over time and are also present in patients with other neurodegenerative disorders and, therefore, not disease specific (Zimmermann and Brockmann 2022). Still, PD patients with above average contribution of neuroinflammation to their disease may be the ones benefiting most from immunomodulatory therapies.

Overall, clinical and molecular subgroups should be more intensively used for the design of targeted clinical trials and could result in more specific therapeutic options for PD patients.

## Device-assisted therapeutic monitoring

The success of symptomatic and disease-modifying therapy is based on regular assessment of symptom burden, side effects, and treatment adherence. Still, 40% of patients with PD in Europe and the US are not evaluated by neurologists or movement disorder specialists (Dorsey et al. 2018), and access to specialized care is particularly limited in rural areas or developing countries (Dorsey and Bloem 2018). In addition, the evaluation of symptoms of PD patients is largely limited to short episodes of in-clinic visits and outpatient consultations as well as patient-completed symptom diaries and subjective reports. Relevant aspects might not be captured in these contacts due to symptom fluctuations, rare occurrences, or relevant differences between supervised assessments and symptoms in daily life (Warmerdam et al. 2020).

Device-assisted digital assessments during inpatients visits can help objectify clinical evaluation, measure treatment response, and help overcome interrater variations. For example, digital motion biomarkers enable measurement of discreet movement disturbances not visible during routine examination (e.g., smoothness of gait and jerk of foot, Kuhner et al. 2019). In parallel to cardinal motor features, machine learning-based speech analyses were able to identify early and mid-stage PD patients with high accuracy (Suppa et al. 2022). To quantify therapy effects, markerless motion capture systems help improve motivation and outcome during neurorehabilitation (Knippenberg et al. 2017).

In addition, remote sensor-based assessments are becoming increasingly important to measure motor symptoms in a natural environment, and real-word studies have shown feasibility and acceptance of tools such as wearable sensors, smartphone apps, and smartwatches (Adams et al. 2021; Bendig et al. 2022; Powers et al. 2021). Most wearable sensors contain accelerometers and gyroscopes and are placed on one or more locations on variable parts of the body such as trunk, upper, or lower extremities. Depending on the device, measurements of bradykinesia, dyskinesia, tremor, gait, falls, or overall physical activity are possible (Ancona et al. 2022) Positive effects on clinical outcome have been shown when experts were supported by wearable sensors in their clinical decision making (Woodrow et al. 2020; Isaacson et al. 2019). Moreover, wearable sensors have also been included in recent trials to measure at home functioning as a secondary outcome (NCT04739423, NCT04380142). To establish the use of wearable sensors into the daily clinical routine, large-scale RCTs validating assessments in real world conditions and giving evidence of benefits of sensor-based assessments over the current clinical standards in terms of therapeutic effects, quality of life, or cost-effectiveness are needed (Del Din et al. 2021). Among others, the Movement Disorder Society has developed a roadmap for the implementation of digital outcome measures to overcome the current limitations (Espay et al. 2019).

## Early diagnosis and biomarker development

There is a growing need for objective biomarkers that allow earlier diagnosis, the quantification of disease-relevant molecular processes, and treatment response for DMTs (The Parkinson Progression Marker Initiative (PPMI) 2011).

Clinical measures (e.g., MDS-UPDRS) provide an estimate of symptom severity and—used in the right setting—valid measure for clinical disease progression as outcome parameters in clinical studies of new DMTs. However, clinical scores are heavily biased by symptomatic effects, do not capture subclinical effects on molecular processes, or effects of potential DMTs in pre-symptomatic subjects. Some efforts in the detection of preclinical symptoms with have been made, and data suggest the potential of wearable accelerometer devices to identify prodromal PD (Schalkamp et al. 2022).

Structural and functional neuroimaging has been used in aiding diagnosis of unclear phenotypes as well as monitoring of therapeutic effects in drug trials as outcome measure. Structural and volumetric MRI findings are subtle in early PD and often not be detectable by conventional MRI. Morphometry analyses revealed reduced gray matter volumes in different cortical and subcortical regions to inconsistent extend. These findings enable differentiation between PD patients and controls or other neurodegenerative disorders with varying accuracy (Saeed et al. 2017). Functional neuroimaging with different radioligands by single photon emission computed tomography (SPECT), however, enables to quantify the integrity of the nigrostriatal system. Occurrence of early non-motor symptoms of PD such as hyposmia and constipation correlate with abnormal dopamine transporter (DAT) binding in ^123^Iβ-CIT SPECT imaging (Jennings et al. 2014). Reduction in DAT binding accompanies disease progression and correlates moderately with MDS-UPDRS scores (Simuni et al. 2018). DAT scan has shown feasible as secondary or exploratory outcome for trials investigating DMTs. In a trial comparing levodopa and pramipexole in early PD patients, patients who received pramipexole showed less reduction in ^123^Iβ-CIT uptake compared to the levodopa group, which also correlated with UPDRS scores (Parkinson Study Group 2002). Patients with normal DAT scans and early non-motor symptoms have been shown to be less likely to convert to PD than patients with changes in DAT scan (Batla et al. 2014; Lee et al. 2021). In addition, nigrostriatal integrity can also be evaluated by positron emission tomography (PET, e.g., by ^18^F-dopa), but PET imaging also harbors promising applications for measuring glucose metabolism (^18^F-FDG), microglia-mediated neuroinflammation (e.g., ^11^C-(*R*)-PK11195), and protein (e.g., aSyn) accumulation (Saeed et al. 2017; Capotosti 2022). With the development of novel imaging markers, in vivo measurements of pathophysiological hallmarks can accompany clinical findings in symptom severity and are crucial for further development of DMTs.

Liquid biomarkers that reflect key pathological hallmarks of PD such as intracellular aggregation and intercellular spread of pathological forms of aSyn (Goedert et al. 2013; Yang et al. 2022) allow stratification of patients by their molecular diagnosis and are important for the success of α-synucleinopathy targeted treatments. So far, the measurement of total aSyn concentrations in the cerebrospinal fluid (CSF) has been unsatisfactory for the diagnosis of PD or as disease progression marker (Ohrfelt et al. 2009; Mollenhauer et al. 2008, 2017; Compta et al. 2015; Majbour et al. 2016; Eusebi et al. 2017). Total aSyn levels likely do not reflect the complex pathophysiology of α-synucleinopathy (Tofaris 2022; Stefanis et al. 2019). A more accurate measurement of disease-specific aSyn was achieved by isolation of neuron-derived extracellular vesicles from serum (Jiang et al. 2020, 2021), even in combination with a seeded aggregation assay (Kluge et al. 2022). The measurement of disease-specific forms of aSyn such as oligomers or aggregates seem most promising to detect α-synucleinopathy even early in disease development. Real‐time quaking‐induced conversion (RT‐QuIC) exploits the aggregation properties of aSyn (Stefanis et al. 2019; Brandel et al. 2015). This assay can detect aSyn aggregation in the CSF of PD and dementia with Lewy bodies (DLB) patients with high sensitivity and specificity (Fairfoul et al. 2016; Groveman et al. 2018; Manne et al. 2019; Bongianni et al. 2019). Importantly, aSyn aggregation properties were detected in prodromal PD patients with clinical syndromes that preceded Parkinsonism or cognitive decline with high sensitivity of over 95%, meaning that RT-QuIC could potentially enable an early diagnosis (Rossi et al. 2020). In the large Parkinson's Progression Markers Initiative cohort of 1123 participants, 99% of PD patients with olfactory disfunction showed seeded aggregation and healthy controls were correctly identified with a specificity of 96% (Siderowf et al. 2023). RT‐QuIC has also been tested in symptomatic and non-symptomatic patients carrying a *LRRK2* mutation, a common genetic risk factor for familial and sporadic PD, and seeding propensity was shown in a subset of patients (Garrido et al. 2019). RT-QuIC could therefore help to identify candidates to receive disease-modifying drugs even in an asymptomatic phase of disease. RT-QuIC from a nasal swab is especially promising since it enables less invasive biosampling (Perra et al. 2021; Stefani et al. 2021). Seeded aggregation assays may also be interesting to detect PD in subjects without initial evidence of dopamine deficit by imaging (Russo et al. 2021). Currently available RT-QuIC essays enable a qualitative analysis with either a negative or positive result. However, quantification of seeded oligomers in a positive sample could additionally help to monitor longitudinal changes over the course of a disease or in response to a treatment (Russo et al. 2021; Majbour et al. 2022).

Disease-unspecific biomarkers reflect downstream effects of pathology and could be proposed for the assessment of disease progression. While neurofilaments are not elevated in CSF and serum in idiopathic PD compared to age matched controls (Hansson et al. 2017; Marques et al. 2019), higher neurofilament levels in PD correlate with progressive motor dysfunction or cognitive decline (Lin et al. 2019; Aamodt et al. 2021). Neurofilament levels are increased in atypical parkinsonian syndromes compared to PD (Herbert et al. 2015; Hall et al. 2012; Hansson et al. 2017; Marques et al. 2019). Therefore, combining neurofilaments with α-synucleinopathy specific biomarkers could be used to stratify patients by individual rate of progression and diagnosis into treatment trials.

In patients with PD-associated dementia, Alzheimer’s like pathology including extracellular β-amyloid plaques as well as intracellular hyperphosphorylated tau (p-tau) deposition are seen in two-thirds of autopsied cases (Jellinger 2012; Smith et al. 2019). Levels of β-amyloid and p-tau181 in CSF have been shown to correlate with cognitive decline in nondemented people and patients with Alzheimer’s dementia, PD dementia, and vascular dementia (Fagan et al. 2007; Skillbäck et al. 2015). Low levels of β-amyloid in CSF have been associated with cognitive impairment in PD, while data for p-tau is inconsistent (Compta et al. 2009, 2013). Most studies show that plasma p-tau levels do not associate with cognitive decline in PD (Lin et al. 2018; Pagonabarraga et al. 2022; Batzu et al. 2022). However, higher plasma p-tau levels seem to predict Alzheimer’s disease pathology in dementia with Lewy bodies and PD dementia (Gonzalez et al. 2022). A prognostic value for cognitive decline in PD has been shown for CSF β-amyloid and neurofilaments (Siderowf et al. 2010; Bäckström et al. 2022). Predicting cognitive decline in PD can be a valuable tool for communicating prognosis to patients as well as clinical management and inclusion into clinical trials.

The accumulation of iron in the substantia nigra is a known feature in PD and increases in the disease course (Lhermitte et al. 1924; Dexter et al. 1989). Early studies on iron levels in PD provided inconclusive evidence on the ability to discriminate between healthy controls and patients with PD (Mariani et al. 2013; Medeiros et al. 2016; Lucio et al. 2019). An elemental cluster including six different elements (including iron) identified PD patients with high sensitivity and specificity (Maass et al. 2018). Further, the iron and ferritin CSF levels show inverse changes in a longitudinal cohort of patients with PD indicating their potential as a progression marker (Maass et al. 2021). Warranting further validation in independent cohorts, change of iron and ferritin levels in response to DMT might, therefore, be used as surrogate marker to evaluate effects on disease progression.

In ALS, NfL already is an established biomarker for disease progression and also predicts phenoconversion in pre-symptomatic mutation carriers (Benatar et al. 2018). This feature is used in the ATLAS trial (Benatar et al. 2022): ATLAS uses pre-symptomatic gene mutation carriers of SOD1 to identify people at risk and submit them to therapy with the antisense oligonucleotide Tofersen. Tofersen was shown to decrease SOD1 and NfL levels in the CSF of treated patients in phase 1–2 and 3 trials (Miller et al. 2020, 2022) although no advantage of decline in the clinical scores was shown within the 24-week follow-up of the phase 3 trial. For the initial studies, however, only symptomatic patients were included. ATLAS now aims at slowing the course of the disease once a threshold of NfL is reached and before clinical symptoms are present. While a biomarker with similar properties is lacking for PD, studies are conducted recruiting patients with premotor symptoms of the disease such as Rapid-eye-movement (REM) sleep behavior disorder (RBD). Even though RBD is not present in all PD patients and some patients develop RBD in later stages of the disease, RBD is considered as one of the earliest and most specific prodromal signs of an α-synucleinopathy (Miglis et al. 2021). Rasagiline (NCT05611372) and idebenone (NCT04152655) are currently assessed for their impact on the progression time from RBD to PD.

Taken together, there are several promising fluid-biomarker candidates covering different aspects of the disease. While aSyn might be a parameter for measuring the pathological hallmark of the disease, NfL, iron, and ferritin levels have the potential to predict the progression of the disease, while β-amyloid and potentially p-Tau are promising to forecast cognitive impairment. While CSF might be the most relevant biomaterial for neurodegenerative diseases and is helpful as diagnostic tool, evaluation of easily accessible biomaterial might be of special relevance for longitudinal assessment. All these biomarkers have the potential to provide a more accurate diagnosis and differentiate subtypes of PD, and therefore will ultimately benefit clinical trial recruitment as well as the selection and monitoring of new therapies. Discovery and validation of new biomarkers will be crucial in refining these processes and thereby aid development of new disease-modifying therapies. In addition, identification of biomarkers predicting phenoconversion even before prodromal signs are present and will be crucial for trials testing disease-modulating substances early in the course of the disease.

## Conclusion

**Fig. 1:**
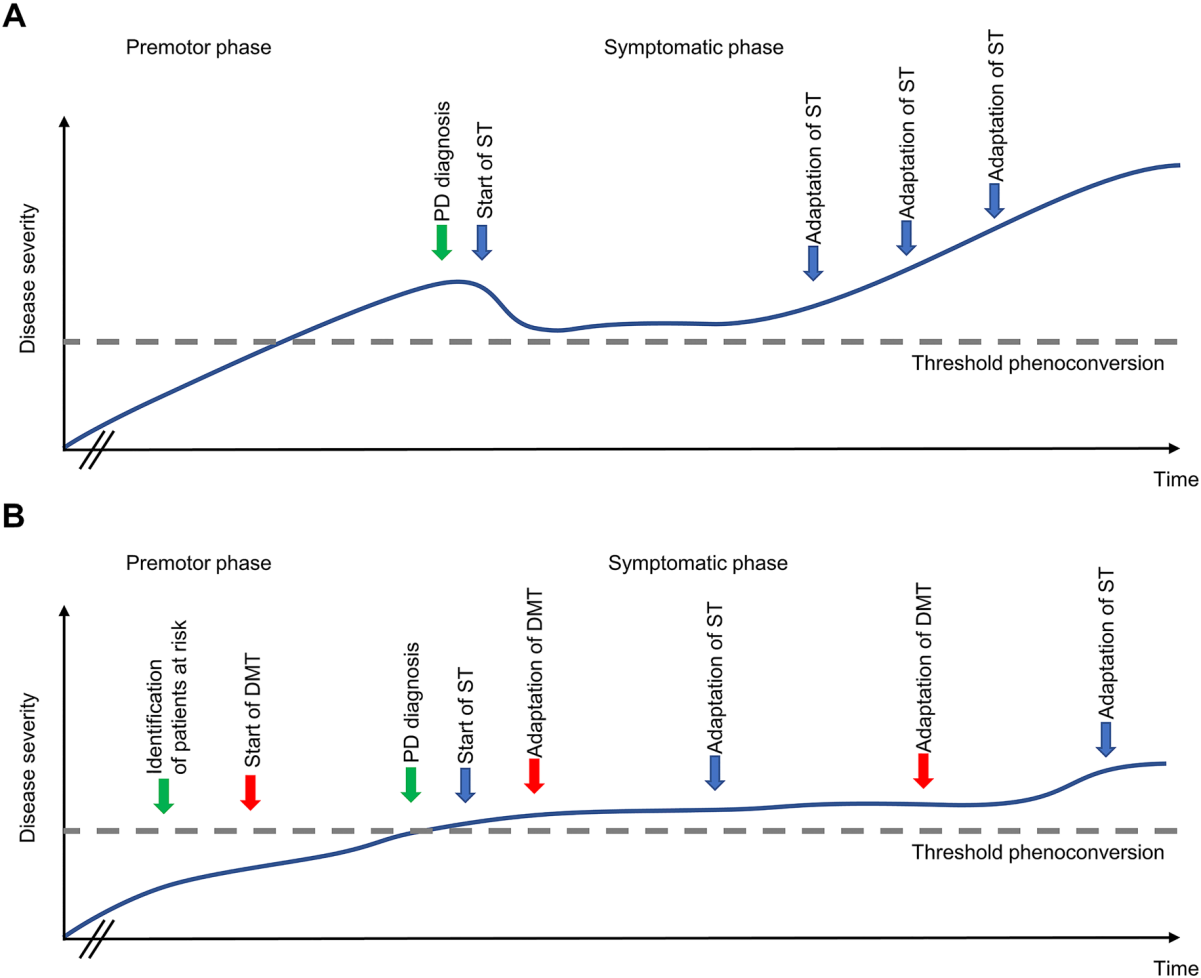
**A** PD therapy currently starts after the development of motor symptoms and diagnosis is made based on clinical symptoms. Symptomatic treatment (ST) is started and initially leads to good symptom control. With increasing disease progression and symptom burden, ST is adapted. **B** In the future, biomarker-based risk stratification will help identify people at risk with subclinical manifestations. Disease-modifying therapy (DMT) will be started in a premotor or prodromal phase, e.g., in a progression marker-based subset of people with high risk of phenoconversion. Clinical PD diagnosis could include a combination of clinical symptoms and biomarkers. Symptomatic therapy (ST) will be started and adapted by symptom severity as experienced by the patient and established clinical scales with the help of digital health applications. DMT will be adapted to the disease stage using biomarker-based stratification

The therapeutic landscape in PD is highly dynamic. Although symptomatic treatments already today allow very good symptom control in earlier disease stages, more advanced stages of PD are still challenging. A big medical need exists in the area of non-motor symptoms, but it is contrasted by a vibrant clinical trial landscape. The development of disease-modifying therapies will fundamentally change the therapeutic landscape in the future. In addition, it can be assumed that with the increasing use of biomarkers, therapy will be more targeted to the individual patient. This will pave the way for future therapies to be applied not only in symptomatic patients, but also to develop therapeutic strategies that start at the pre-symptomatic stage and can thus delay the onset of the disease and mitigate its progression (Fig. 1).

## Data Availability

Data sharing not applicable to this article as no datasets were generated or analysed for this article.

## Abbreviations

aSyn: Alpha-synuclein
CSAI: Continuous subcutaneous apomorphine infusion
DBS: Deep brain stimulation
DMT: Disease-modifying therapy
EMA: European Medicines Agency
FDA: Food and Drug Administration
GBA: Glucocerebrosidase
GPI: Globus pallidus internus
LCIG: Levodopa–carbidopa intestinal gel
LECIG: Levodopa–entacapone–carbidopa intestinal gel
LRRK2: Leucine-rich repeat kinase 2
MRgFUS: MR-guided focused ultrasound
PD: Parkinson’s disease
PRS: Polygenic risk scores
RCTs: Randomized controlled trials
SSRI: Selective serotonin reuptake inhibitor
SNRI: Serotonin norepinephrine reuptake inhibitor
TCA: Tricyclic antidepressant
STN: Subthalamic nucleus
VIM: Nucleus ventralis intermedius thalami
